# Machine learning identifies lipid-associated genes and constructs diagnostic and prognostic models for idiopathic pulmonary fibrosis

**DOI:** 10.1186/s13023-025-03876-0

**Published:** 2025-07-10

**Authors:** Xingren Liu, Junmei Song, Shujin Guo, Yi Liao, Jun Zou, Liqing Yang, Caiyu Jiang

**Affiliations:** 1 Department of Respiratory and Critical Care Medicine, School of Medicine, Sichuan Provincial People’s Hospital, University of Electronic Science and Technology of China, Chengdu, China; 2 Ultrasound in Cardiac Electrophysiology and Biomechanics Key Laboratory of Sichuan Province, Sichuan Clinical Research Center for Cardiovascular Disease, Sichuan Provincial People’s Hospital, University of Electronic Science and Technology of China, Chengdu, China; 3 Department of Cardiovascular Ultrasound & Noninvasive Cardiology, Sichuan Provincial People’s Hospital, University of Electronic Science and Technology of China, Chengdu, China; 4 Department of Health Management & Institute of Health Management, School of Medicine, Sichuan Provincial People’s Hospital, University of Electronic Science and Technology of China, Chengdu, China

**Keywords:** Idiopathic pulmonary fibrosis, Lipid, Diagnosis, Prognosis

## Abstract

**Objective:**

Emerging evidence suggests a potential relationship between lipid metabolism and idiopathic pulmonary fibrosis (IPF). This study aimed to identify lipid-related genes implicated in IPF pathogenesis.

**Methods:**

Lipid-associated genes were retrieved from the GeneCards database and analyzed using unsupervised consensus clustering to classify IPF samples. Weighted gene co-expression network analysis (WGCNA) was performed on the identified clusters to determine core modules and hub genes associated with IPF. Machine learning algorithms were applied to these hub genes to construct diagnostic and prognostic models, which were validated across multiple datasets. Single-cell sequencing was used to investigate the distribution of potential pathogenic genes, and their functional roles were further validated through cellular experiments.

**Results:**

Two distinct clusters were identified, showing significant differences in lung function parameters and fibrosis-related gene expression. WGCNA revealed that the blue module was strongly associated with IPF and served as the core module. Genes from this module were used to construct diagnostic and prognostic models, which demonstrated strong predictive performance across multiple validation datasets. Single-cell sequencing revealed that KLF4 was highly expressed in lung epithelial cells. Functional assays indicated that knockdown of KLF4 did not affect the proliferation of human alveolar type II epithelial cells but significantly enhanced their migratory capacity, thereby promoting the fibrotic process.

**Conclusion:**

This study successfully constructed lipid-related diagnostic and prognostic models for IPF and identified KLF4 as a potential causative gene. These findings provide a foundation for further exploration of lipid metabolism in IPF pathogenesis and potential therapeutic strategies targeting KLF4.

**Supplementary Information:**

The online version contains supplementary material available at 10.1186/s13023-025-03876-0.

## Background

Idiopathic pulmonary fibrosis (IPF) is an interstitial lung disease of unknown origin, characterized by abnormal alveolar repair that triggers excessive myofibroblast proliferation and extracellular matrix (ECM) accumulation, leading to fibrosis and functional impairment of lung tissue [1, 2]. A literature review of population-based studies from January 2009 to April 2020 estimated the global incidence and prevalence of IPF [3]. Adjusted incidence rates ranged from 0.09 to 1.30 per 10,000 people in Europe, North America, and Asia-Pacific countries, with South Korea showing the highest figures. Adjusted prevalence rates ranged from 0.33 to 4.51 per 10,000 people, with IPF considered a rare disease in most countries except South Korea [3]. The prognosis for patients with IPF is poor. A review that included multiple cohort studies and randomized controlled trials reported mortality rates of 12%, 38%, and 69% at 1–2 years, 2–5 years, and ≥ 5 years, respectively, with a mean overall survival of 4 years for studies with a 10-year follow-up [4]. It is clear that IPF continues to pose a serious public health burden.

The lungs are abundant in lipids, with lipid metabolism being particularly active, especially in the distal alveolar regions. This includes surfactant, which is primarily composed of lipids, with a lipid content as high as 90%. Surfactant plays a crucial role in reducing surface tension at the air-liquid interface, preventing alveolar collapse at the end of exhalation, and maintaining alveolar stability [5]. Lipids have also been shown to have potential associations with IPF [6], suggesting their involvement in the disease’s pathogenesis. A study on a mouse model of pulmonary fibrosis revealed abnormal accumulation of ECM lipid components in bronchoalveolar lavage fluid (BALF), which further suggests that lipid metabolism dysregulation may play a key role in ECM remodeling and the pathogenesis of IPF [7]. In the lungs of IPF patients, the expression of low-density lipoprotein receptor (LDLR) is reduced, while plasma LDL levels are elevated. Compared to normal mice, LDLR knockout mice exhibit higher LDL levels, with fibrosis developing earlier and more severely. Additionally, in vitro experiments have shown that LDL induces cell apoptosis and the production of transforming growth factor-β1 (TGF-β1), thereby exacerbating the progression of fibrosis [8]. This further suggests that dysregulation of lipid metabolism may play a critical role in ECM remodeling and the pathogenesis of IPF.

This study utilized data from the Gene Expression Omnibus (GEO) database to systematically analyze genes closely associated with lipid metabolism through a range of bioinformatics approaches. Potential core genes relevant to IPF were identified and further investigated. Prognostic and predictive models for IPF patients were constructed based on these core genes, providing novel insights for disease evaluation. To validate the findings derived from bioinformatics analyses, cellular experiments were conducted to examine the expression profiles of lipid metabolism-related genes in alveolar epithelial cells and to evaluate their impact on fibrosis development. By combining computational analyses with experimental validation, the study offers a valuable framework for identifying potential predictive biomarkers and understanding the molecular mechanisms underlying IPF. These findings aim to contribute to the development of improved diagnostic and therapeutic strategies for the disease.

## Methods

### Data processing

Relevant sequencing data were downloaded from the GEO database, including samples from IPF patients (lung tissue, BALF, and blood) and healthy control groups. Some datasets also provided clinical and prognostic information. The raw data were standardized using the limma package [9], followed by log2 transformation. Probe names were converted into gene names based on platform annotations, and for genes corresponding to multiple probes, the average expression value was calculated. The sequencing datasets utilized in this analysis include GSE47460 [10], GSE32537 [11], GSE53845 [12], and GSE150910 [13], all of which comprise sequencing data from lung tissue samples of IPF patients and healthy controls. Additionally, three datasets containing prognostic information for IPF patients were included: GSE70866 [14] (BALF) and two blood-derived datasets, GSE28221 [15, 16] and GSE93606 [17]. GSE70866 provided overall survival (OS) data, GSE28221 included transplant-free survival (TFS) data, and GSE93606 offered progression-free survival (PFS) information.

### Unsupervised consensus clustering analysis

The GSE47460 dataset, which includes detailed clinical information such as gender, age, and partial lung function indicators, was selected for this clustering analysis due to its comprehensive data. Genes related to lipids were identified using the GeneCards database [18], and unsupervised consensus clustering analysis was performed based on these genes.

### Immune infiltration analysis

Cell-type Identification By Estimating Relative Subsets Of RNA Transcripts (CIBERSORT) [19] was utilized to calculate the relative proportions of 22 immune cell types in each sample using the LM22 signature matrix. The gene expression data were input into the algorithm, which estimated the immune cell composition through linear support vector regression.

### Weighted Gene Co-Expression Network Analysis (WGCNA)

WGCNA [20] was applied to identify gene modules associated with specific traits using a gene expression matrix. Genes with the top 25% variance were selected, normalized, and processed to highlight biologically relevant features. Sample clustering was conducted to identify outliers, which were subsequently excluded, and trait data were incorporated into the analysis. The optimal soft-threshold power was determined to ensure scale-free topology, and adjacency and topological overlap matrices (TOM) were constructed to evaluate gene correlations. Gene modules were identified through hierarchical clustering and dynamic tree cutting, with similar modules merged based on eigengene correlations. Relationships between module eigengenes and clinical traits were assessed, and significant modules underwent further investigation using gene significance (GS) and module membership (MM) calculations. The findings were visualized through dendrograms, heatmaps, and scatter plots. Key outputs, including the filtered gene matrix, module-trait associations, and gene-level GS and MM data, were exported for subsequent analysis.

### Functional enrichment analysis

Functional enrichment analysis was performed to explore the biological significance of the identified genes and modules. Disease Ontology (DO) enrichment analysis [21] was conducted to link genes to disease categories, while Gene Ontology (GO) analysis [22] was applied to assess the genes’ involvement in biological processes, molecular functions, and cellular components. Kyoto Encyclopedia of Genes and Genomes (KEGG) pathway analysis [23] was utilized to identify enriched signaling and metabolic pathways. Additionally, Gene Set Variation Analysis (GSVA) was employed to evaluate the activity of predefined gene sets across samples, providing insights into pathway-level variations.

### Construction and analysis of the PPI network

The analysis started with retrieving protein-protein interaction (PPI) data for the target gene set from the STRING database [24]. The PPI network was subsequently constructed, and gene degree analysis was performed using Cytoscape software [25].

### Development and validation of a diagnostic model

As previously described [26], multiple machine learning algorithms, either independently or in combination, were employed to construct the diagnostic model. The GSE47460 dataset was utilized as the training set, while GSE32537, GSE53845, and GSE150910 served as validation datasets. The model’s predictive performance was evaluated using Receiver Operating Characteristic (ROC) curves, and its accuracy was further demonstrated through the use of confusion matrices. The methodological approach aligns with established practices detailed in prior work, ensuring the reliability and reproducibility of the model construction process.

### Development and validation of a prognostic model

As outlined in prior research [26], multiple machine learning algorithms, either individually or in combination, were utilized to construct the prognostic model. The GSE70866 dataset was designated as the training set, while GSE28221 and GSE93606 were used for validation. Time-dependent Receiver Operating Characteristic (ROC) curves were employed to evaluate the predictive performance of the model. Additionally, the performance of this model was compared against other published models to highlight its effectiveness and potential advantages.

### Single-cell sequencing analysis

The GSE128033 dataset was utilized for single-cell RNA sequencing analysis. Single-cell RNA sequencing data were processed and analyzed using the Seurat package [27]. Cells with fewer than 200 or more than 10,000 detected features, total RNA counts below 1,000, or mitochondrial and ribosomal gene content exceeding 20% were excluded to ensure data quality. Multiple datasets were iteratively integrated to generate a consolidated Seurat object for downstream analysis. The data were normalized using the LogNormalize method, followed by dimensionality reduction of highly variable genes through Principal Component Analysis (PCA) and Uniform Manifold Approximation and Projection (UMAP). Batch effects were corrected using Harmony [28], and clustering was performed at the optimal resolution based on shared nearest neighbor graphs. SingleR was applied to annotate the clusters and determine cell types. Visualization of cell types and gene distributions was achieved through violin plots, feature plots, and heatmaps, while cell type proportions were calculated and displayed using bar plots.

### Cell culture

Primary human alveolar type II (AT II) epithelial cells (Product ID: CP-H209), human bone marrow-derived macrophages (Product ID: CP-H186), and human lung fibroblasts (Product ID: CP-H011) were purchased from Procell Co., Ltd. (Wuhan, China). The cells were cultured in their respective manufacturer-recommended media under standard conditions.

### Cell proliferation assay using CCK-8

Cell proliferation was assessed using the Cell Counting Kit-8 (CCK-8, Beyotime, Cat# C0043) according to the manufacturer’s instructions. Cells were seeded into 96-well plates at a density of 1,000 cells per well in 100 µL of complete culture medium. Plates were incubated at 37 °C in a humidified atmosphere containing 5% CO₂. At 0, 24 and 48 h, 10 µL of CCK-8 reagent was added to each well. After a 2-hour incubation, the absorbance at 450 nm was measured using a microplate reader. All experimental conditions were run in triplicate. The optical density values reflected the relative number of viable cells and were used to evaluate proliferative activity. Precautions were taken to avoid air bubbles and ensure consistent mixing before measurement.

### 5-Ethynyl-2’- Deoxyuridine (EDU)

The BeyoClick™ EdU-555 Cell Proliferation Assay Kit (Beyotime, Shanghai, China) was used to assess proliferation. Cells were seeded in 6-well plates and treated after overnight incubation. A 10 µM EdU solution was prepared, added to the wells, and incubated for 2 h. After fixation, permeabilization, and Click reaction staining, Hoechst 33,342 was used for nuclear staining if needed. Fluorescence microscopy or flow cytometry measured proliferation via Azide 555 fluorescence intensity.

### Scratch assay for evaluating cell migration

The scratch assay was utilized to assess cell migration. Preparations involved preheating PBS, high-glucose DMEM, and trypsin at 37 °C, alongside UV sterilization of the workspace for 30 min. Cells (1 × 10⁶) were seeded into each well of a 6-well plate according to the experimental design. The next day, the medium was replaced with 2% serum medium, and scratches were created using a 200 µL pipette tip by gently drawing four lines across the well bottom. After rinsing twice with PBS, fresh 2% serum medium was added, and images were captured at 0 h. The plate was incubated at 37 °C with 5% CO2, and cell migration was observed through images taken at intervals of 0 and 24 h.

### Real-Time Quantitative Polymerase Chain Reaction (RT-qPCR)

Cell samples were collected from 6-well plates at 80% confluence. Cells were centrifuged at 2000 rpm for 5 min, and the supernatant was removed. The cell pellet was resuspended in 1 mL of Trizol, mixed thoroughly, and incubated at room temperature for 5 min before transferring to a new 1.5 mL EP tube. Total mRNA was extracted using the Trizol RNA extraction kit following the manufacturer’s instructions. Reverse transcription and PCR were performed, with GAPDH used as the reference gene. Primer sequences are provided in Supplementary Table 1.

### Western Blotting (WB)

Western blotting was performed using cell samples collected from 6-well plates at 80% confluence. After lysis with 150 µL of 1× Lysis Buffer and centrifugation at 12,000 g, the supernatant was processed for SDS-PAGE. Proteins were separated at 30 mA for 2 h and transferred to PVDF membranes at 4 °C and 400 mA for 120 min. Membranes were blocked with 5% skim milk, incubated overnight with primary antibodies at 4 °C, and washed with TBST. Secondary antibodies (Licor IRDye 800CW) were applied for 2 h at room temperature, followed by TBST washes. Detection was performed using the Tanon-5200 chemiluminescence system.

### Statistical analysis

This study utilized R software (version 4.3.1) for data analysis. Spearman’s rank correlation was employed to evaluate associations between variables. Survival differences were examined using Kaplan-Meier curves with the log-rank test, and the surv_cutpoint function was applied to determine the optimal threshold for survival group stratification.The Wilcoxon rank-sum test was applied for two-group comparisons, while the Kruskal-Wallis test was used for multi-group analyses. A significance level was set at a two-sided *P*-value of less than 0.05.

## Results

### Unsupervised consensus clustering reveals two lipid-associated clusters

**Fig. 1:**
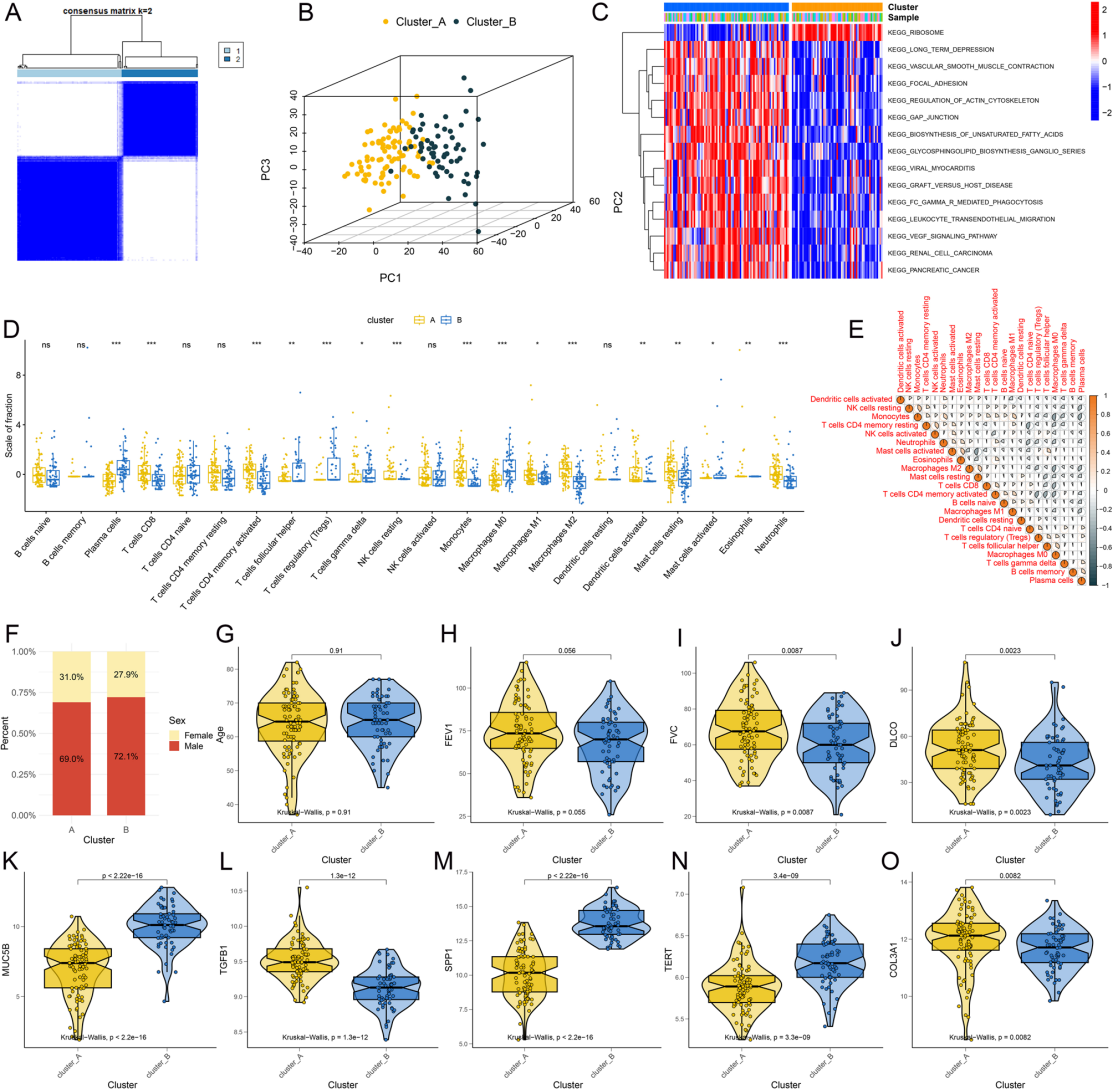
Results of Unsupervised Consensus Clustering Analysis. (**A**). The consensus matrix heat map illustrates the consensus values for each cluster, represented by a gradient ranging from white to blue. (**B**). The principal component analysis plot indicates a clear distinction between Cluster A and Cluster B.(**C**). Heatmap showcasing GSVA enrichment score comparisons for differentially expressed pathways across the two clusters. (**D**). Comparison of immune cell differences between the two distinct clusters. (**E**). Correlation analysis among different immune cells. (**F**). Distribution of different genders among patients in Cluster A and Cluster B. Differences in age (**G**), FEV1 (**H**), FVC (**I**), and DLCO (**J**) parameters between Cluster A and Cluster B. Differences in the expression of MUC5B (**K**), TGFB1 (**L**), SPP1 (**M**), TERT (**N**), and COL3A1 (**O**) genes between Cluster A and Cluster B

After preprocessing the GSE47460 dataset and removing samples with incomplete clinical information, sequencing data from 145 lung tissue samples of IPF patients were included in the analysis. As shown in Fig. 1A, unsupervised consensus clustering analysis based on lipid-related genes obtained from the GeneCards database identified two distinct clusters, labeled Cluster A and Cluster B. Principal Component Analysis (PCA) indicated a clear distinction between the two clusters (Fig. 1B). GSVA analysis revealed significant differences in pathway activity between the clusters. Cluster A showed higher activity in pathways related to ribosome processes and cytoskeletal regulation, whereas Cluster B exhibited elevated activity in pathways associated with fatty acid biosynthesis and immune-related processes, such as leukocyte migration and VEGF signaling (Fig. 1C). Immune infiltration analysis highlighted statistically significant differences in immune cell populations, including Plasma cells, CD8 + T cells, and activated CD4 + memory T cells, between the two clusters (Fig. 1D). Additionally, correlations between certain immune cell types were observed (Fig. 1E), such as a positive correlation between Monocytes and resting NK cells and a negative correlation between activated and resting Mast cells. Differences in clinical characteristics between the two clusters were also analyzed (Figs. 1F-O). While no significant differences were observed in gender ratio, age, or FEV1, significant differences were found in FVC and DLCO. Finally, comparisons of potential IPF-related genes revealed significant differences in the expression of MUC5B, TGFB1, SPP1, TERT, and COL3A1 between the two clusters.

### WGCNA

**Fig. 2:**
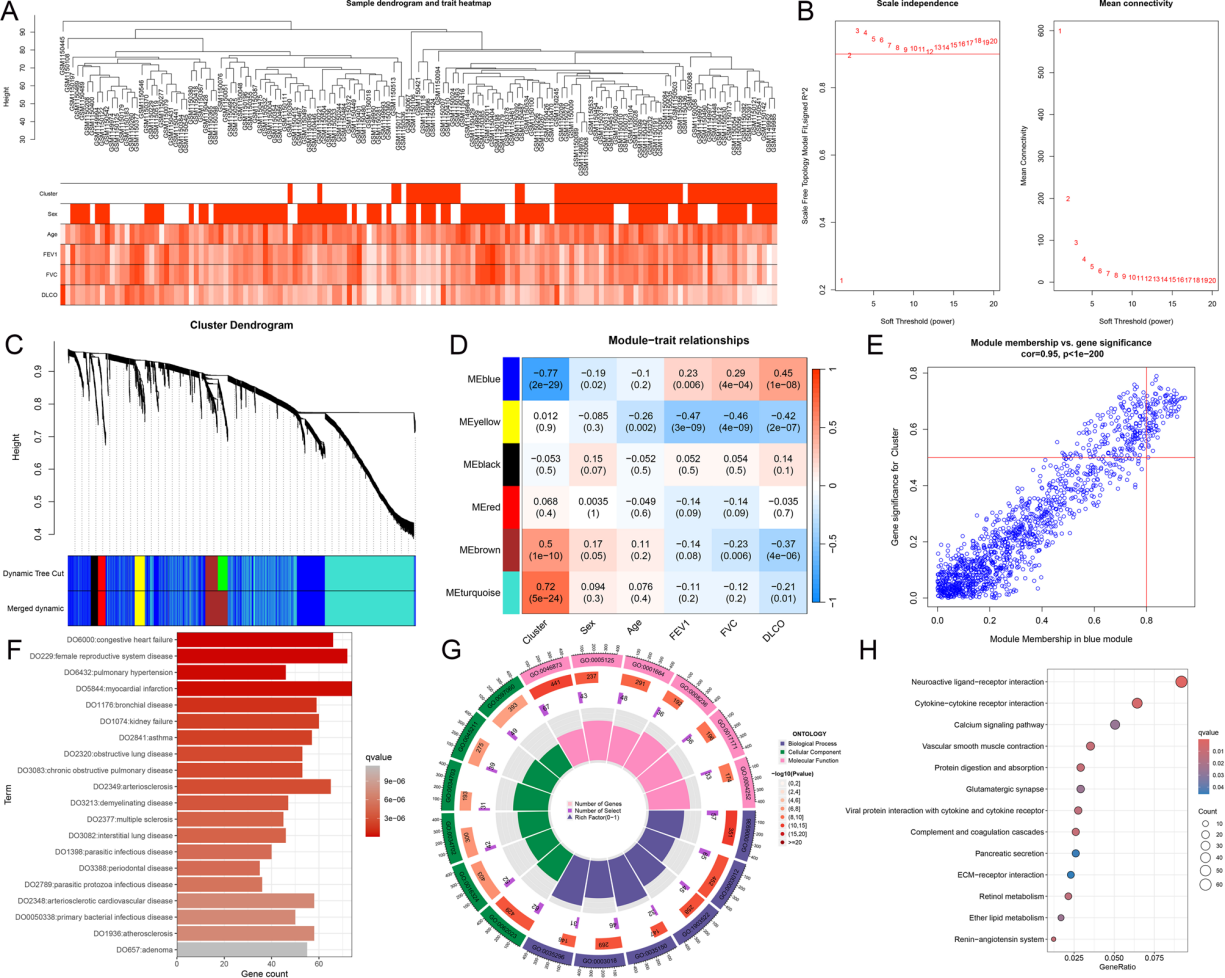
Results of WGCNA. (**A**). Hierarchical clustering dendrogram of IPF patient samples, along with their associated clinical data. (**B**). Selection of the soft-thresholding power in WGCNA, showing the scale-free topology index analysis for different soft-thresholding powers (β) on the left and the mean connectivity analysis for varying soft-thresholding powers on the right. (**C**). Gene dendrogram constructed using the topological overlap matrix measure, accompanied by a color band indicating the module assignments derived from automatic single-block analysis. (**D**). Heatmap showing the relationship between module eigengenes and clinical traits. (**E**). Scatter plot depicting the relationship between gene significance and module membership within the blue module. (**F**). Disease Ontology analysis of the blue module. (**G**). Gene Ontology analysis of the blue module. (**H**). Kyoto Encyclopedia of Genes and Genomes analysis of the blue module

Prior to performing WGCNA, it was confirmed that there were no missing values in the dataset. A hierarchical clustering analysis of all samples was then conducted, with clustering heights ranging from 30 to 90, indicating no outliers or anomalies (Fig. 2A). As shown in Fig. 2B, the scale-free topology fit index first exceeded 0.9 at a soft threshold (β) value of 4, which was selected as the optimal threshold. Based on this, the gene adjacency matrix was transformed into a topological overlap matrix (TOM), and a dendrogram was constructed using 1-TOM as a measure of dissimilarity. Dynamic tree cutting was applied to identify gene modules (Fig. 2C). The blue module demonstrated the strongest correlation with the clusters and was significantly positively associated with lung function indices (Fig. 2D). GS, defined as the correlation between individual genes and traits of interest, was used to quantify the relationship between genes and the phenotypes. MM was defined as the correlation between a module eigengene and gene expression, quantifying the similarity between individual genes and each module. A scatter plot of GS versus MM in the blue module revealed a strong positive correlation between these metrics (Fig. 2E), further emphasizing the functional relevance of this module.

### Functional enrichment analysis

The blue module underwent functional enrichment analysis, revealing its significant association with various biological processes and pathways. DO analysis (Fig. 2F) highlighted links to diseases such as congestive heart failure, pulmonary hypertension, chronic obstructive pulmonary disease, and asthma, suggesting its relevance to cardiovascular and pulmonary conditions. GO analysis (Fig. 2G) indicated enrichment in biological processes like actin cytoskeleton regulation and immune response, with cellular components linked to extracellular matrix organization and molecular functions such as receptor and calcium ion binding. KEGG pathway analysis (Fig. 2H) identified pathways including neuroactive ligand-receptor interaction, cytokine-cytokine receptor interaction, vascular smooth muscle contraction, and ECM-receptor interaction. These results suggest the blue module genes play a critical role in immune regulation, metabolic processes, and extracellular matrix signaling, which are central to disease progression and pathology.

### Development and validation of a diagnostic model

**Fig. 3:**
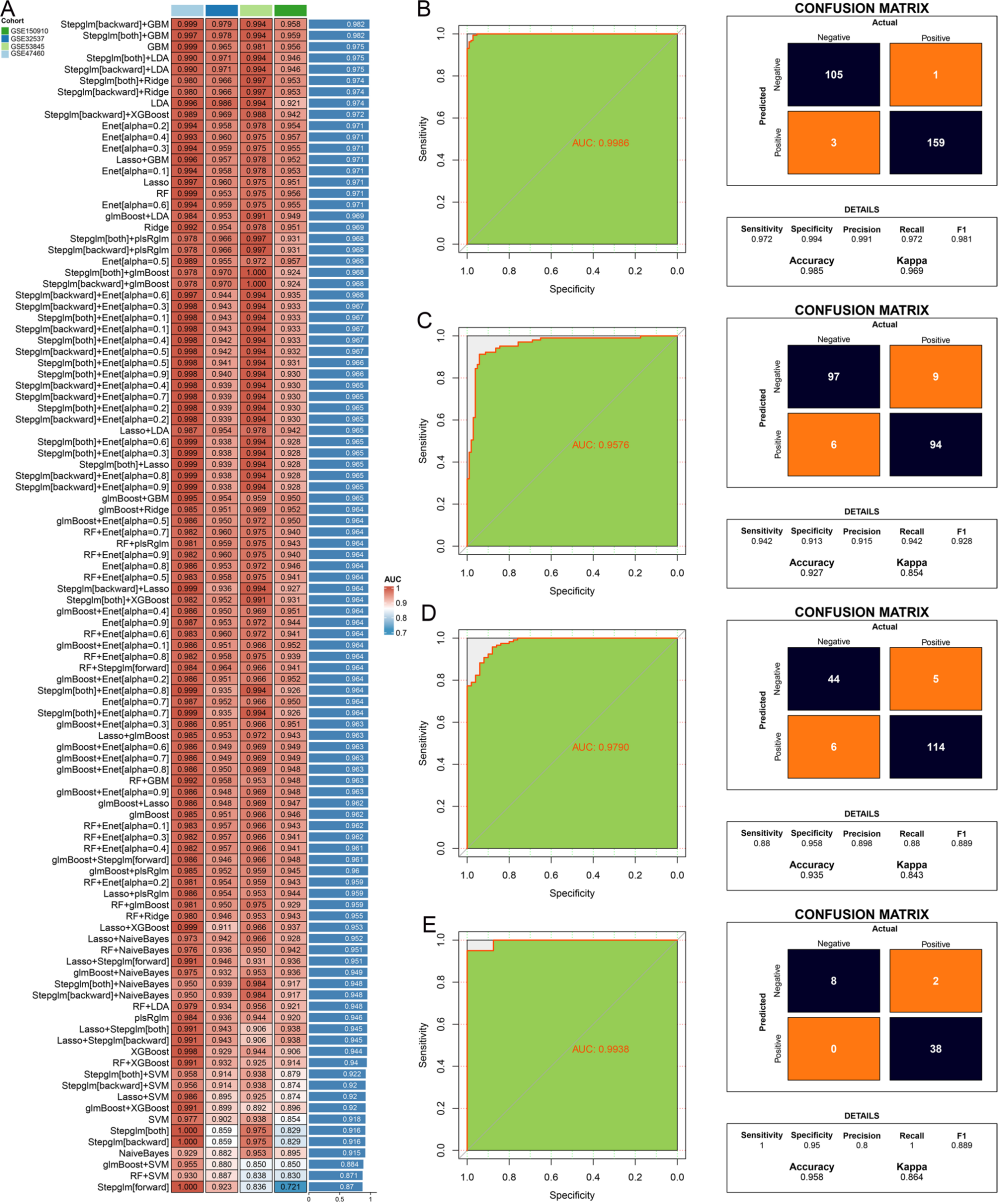
Construction and validation of diagnostic models. (**A**). AUC values of models constructed using different machine learning algorithms across various datasets. ROC curves and confusion matrices for (**B**) GSE47460, (**C**) GSE150910, (**D**) GSE32537, and (**E**) GSE53845

Genes from the blue module with both GS and MM greater than 0.4 were selected as lipid-related core genes (Supplementary Table 2) associated with IPF for subsequent machine learning analysis. A total of 104 machine learning algorithm configurations were employed to construct diagnostic models, with their respective AUC values presented in Fig. 3A. Among these, the diagnostic model built using the Stepglm[backward] + GBM algorithm (Supplementary Table 3), consisting of 15 genes, achieved the highest average AUC across four datasets. In both the training and three validation sets, the AUC exceeded 0.95, indicating excellent predictive performance. Detailed AUC values and confusion matrices for each dataset are shown in Figs. 3B–E.

**Fig. 4:**
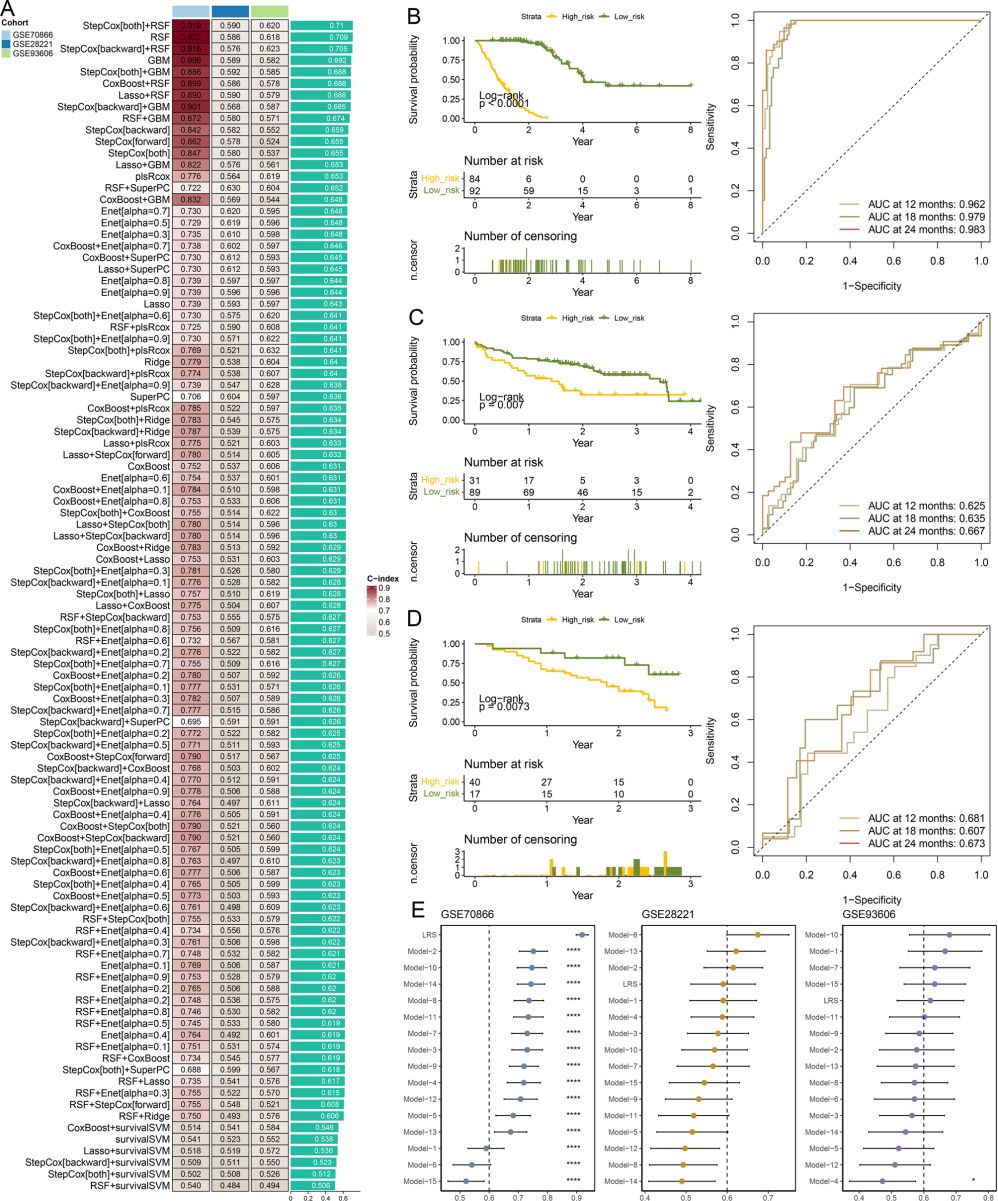
Construction and validation of prognostic models. (**A**). C-index of models constructed using different machine learning algorithms across various datasets. Survival curves and time-dependent ROC curves for (**B**) GSE70866, (**C**) GSE28221, and (**D**) GSE93606. (**E**). Comparison of the prognostic model in this study with previously published models

### Development and validation of a prognostic model

A total of 101 machine learning algorithm configurations were utilized to construct prognostic models and calculate the lipid-related riskscore (LRS) for each sample. The c-index values for all algorithms are presented in Fig. 4A. Among these, the prognostic model built using the StepCox[both] + RSF algorithm, consisting of 10 genes (Supplementary Table 4), achieved the highest average c-index across three datasets. As shown in Figs. 4B–D, patients with high LRS exhibited significantly worse prognosis compared to those with low LRS, with time-dependent ROC curves further illustrating the predictive performance in the same figures. Additionally, the model’s prognostic ability was compared with 15 previously published models [29–43] (Figs. 4E). In the GSE70866 dataset, the proposed model outperformed all other models. Although its performance in the two validation datasets did not exceed that of all other models, it remained among the top-performing approaches.

### Single cell sequencing results

**Fig. 5:**
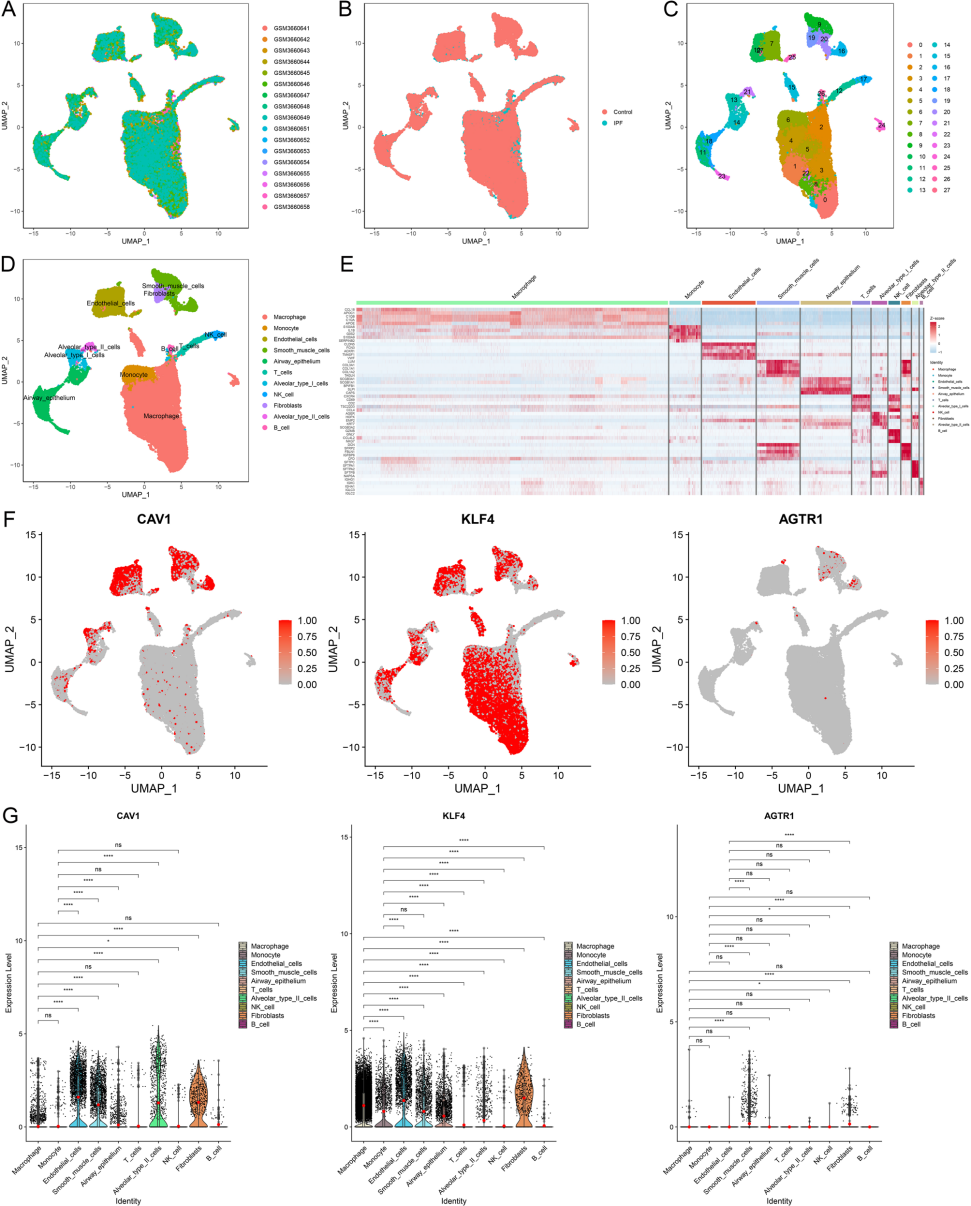
Single-cell sequencing results. (**A**) Uniform Manifold Approximation and Projection (UMAP) visualization of all samples. (**B**) UMAP plots highlighting differences between control and IPF samples. (**C**) UMAP representation showing 28 distinct cell clusters. (**D**) Classification and annotation of 9 unique cell types. (**E**) Heatmap illustrating the top 5 marker genes for each of the 9 cell types. (**F**) Feature plots depicting the expression patterns of 3 genes across the identified cell types. (**G**) Differential expression of 3 hub genes among the 9 cell types

To ensure data consistency and minimize technical bias, sample GSM3660650 was excluded due to a lower feature count (33,538) compared to other samples (33,694). The remaining single-cell RNA sequencing data underwent rigorous quality control, including the removal of doublets, dead cells, and empty droplets. UMAP analysis demonstrated a clear separation between IPF and healthy control samples (Fig. 5A and B). Cell annotation using SingleR identified 28 clusters (Fig. 5C), which were categorized into nine major cell types: macrophages, monocytes, endothelial cells, smooth muscle cells, epithelial cells, T cells, NK cells, fibroblasts, and B cells (Supplementary Fig. 1B). Epithelial cells were further subclassified based on canonical marker genes (Supplementary Fig. 1A), resulting in ten refined cell types: macrophages, monocytes, endothelial cells, smooth muscle cells, airway epithelium, AT II epithelial cells, T cells, NK cells, fibroblasts, and B cells (Fig. 5D). A heatmap was generated to illustrate the expression patterns of representative marker genes across these cell populations (Fig. 5E). The expression of three hub genes identified from PPI network analysis, CAV1, KLF4, and AGTR1 (Supplementary Table 5), was examined across cell types using single-cell data (Fig. 5F). As shown in Fig. 5G, CAV1 was widely expressed, with elevated levels in fibroblasts, AT II epithelial cells, and endothelial cells. KLF4 showed high expression in fibroblasts and epithelial cells, but was low in T cells and NK cells. AGTR1 exhibited low overall expression, with limited presence in macrophages and smooth muscle cells. The proportion of AT II epithelial cells with low KLF4 expression was markedly higher in the IPF group than in controls. This pattern was not observed in fibroblasts or macrophages (Supplementary Fig. 1C). Based on its cell type–specific expression and disease-associated downregulation in AT II epithelial cells, KLF4 was selected for further functional analysis. To evaluate lipid metabolic activity, a curated gene set from the GeneCards database was used to calculate module scores with the AddModuleScore function. Compared to controls, IPF samples displayed significantly lower lipid metabolism scores (Supplementary Fig. 2A). Among cell types, macrophages exhibited the highest lipid activity, followed by fibroblasts and AT II epithelial cells. In contrast, T cells, NK cells, and B cells showed the lowest scores (Supplementary Fig. 2B). These results indicate a distinct pattern of lipid metabolic reprogramming across cell types in the IPF lung environment.

### KLF4 knockdown enhances fibrosis and migration in AT II epithelial cells

**Fig. 6:**
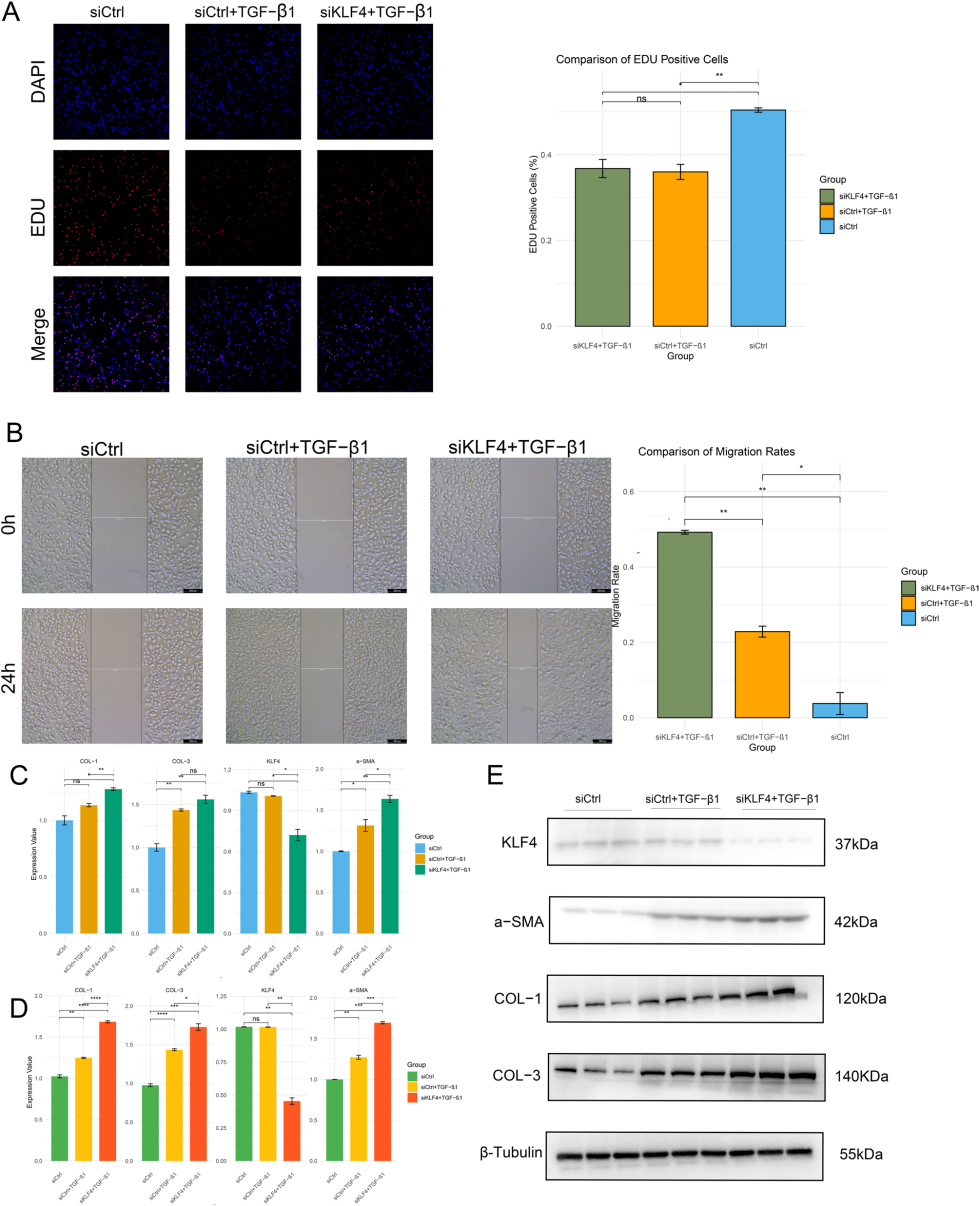
Cell experiment results. (**A**). EdU incorporation assays revealed that TGF-β1 promoted human alveolar type II epithelial cells proliferation, but this effect was not further enhanced by KLF4 knockdown. (**B**). Scratch assays indicated that TGF-β1 enhanced the migratory capacity of human alveolar type II epithelial cells, which was further amplified by KLF4 knockdown. (**C**). RT-qPCR results. (**D**). Western blot results. (**E**). Western blot band diagram

The potential profibrotic role of KLF4 in AT II epithelial cells was investigated through relevant cellular experiments. The cells were divided into three groups: a control group treated with blank siRNA, a group treated with TGF-β1, and a group treated with siRNA to knock down KLF4 followed by TGF-β1 treatment. As shown in Fig. 6A, the addition of TGF-β1 inhibited the growth of AT II epithelial cells, while knocking down KLF4 had no significant effect on cell growth. The scratch assay (Fig. 6B) revealed that TGF-β1 enhanced the migratory ability of AT II epithelial cells, which was further increased after KLF4 knockdown. Figures 6C-E demonstrate that PCR and Western blotting confirmed the successful knockdown of KLF4 in AT II epithelial cells using siRNA. The addition of TGF-β1 promoted fibrosis in AT II epithelial cells, and fibrosis was further exacerbated after KLF4 knockdown.

### KLF4 knockdown enhances the proliferation and migration of macrophages and fibroblasts

**Fig. 7:**
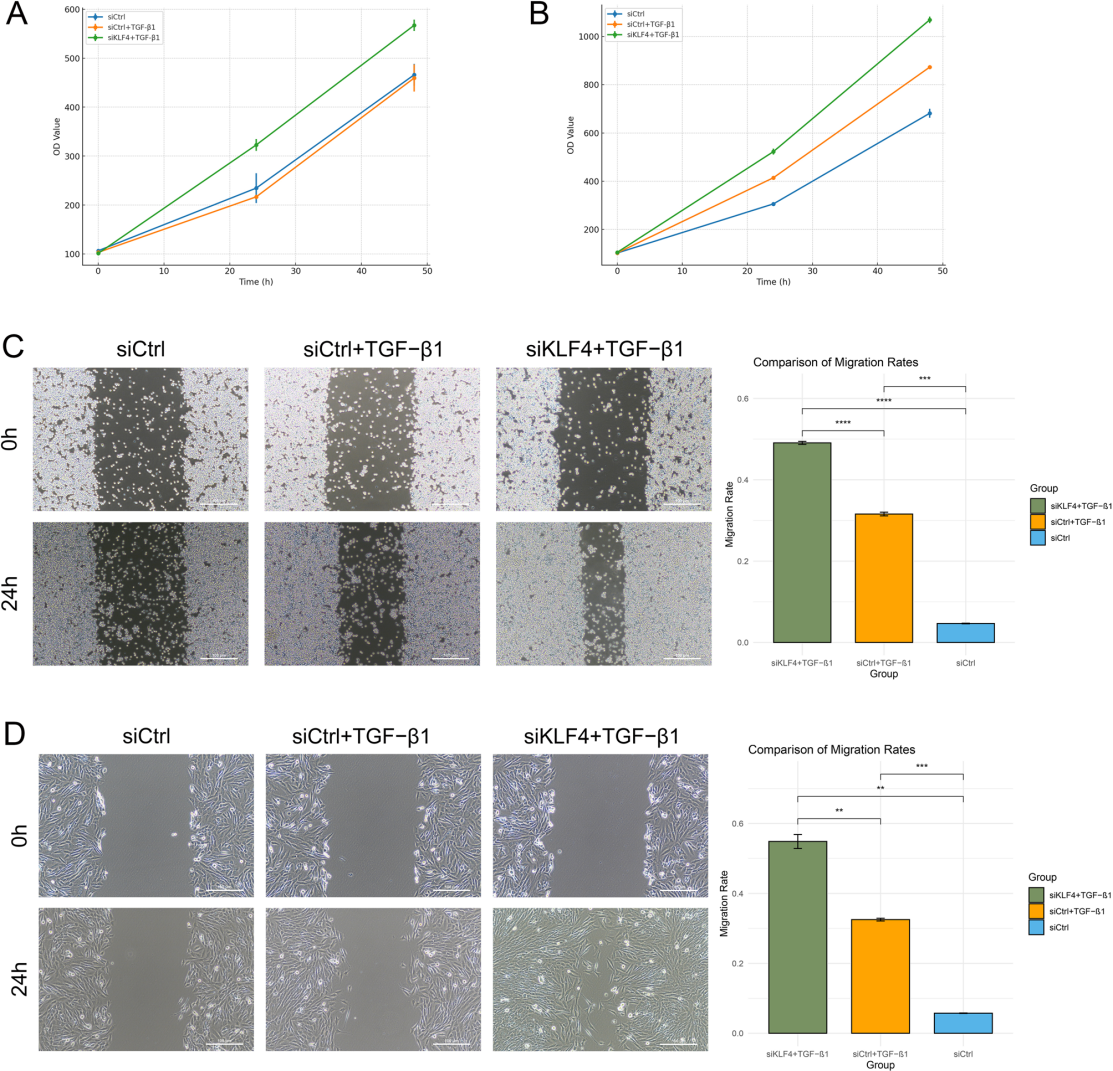
Cell experiment results. (**A**) CCK-8 assay showing the proliferation curve of macrophages. (**B**) CCK-8 assay showing the proliferation curve of fibroblasts. (**C**) Wound healing assay results for macrophages. (**D**) Wound healing assay results for fibroblasts

To assess the functional impact of KLF4, CCK-8 and wound healing assays were conducted in macrophages and fibroblasts. In both cell types, the siKLF4 + TGF-β1 group exhibited significantly increased proliferation compared to control groups, as shown by CCK-8 assays over 48 h (Fig. 7A and B). Wound healing assays further demonstrated that KLF4 knockdown enhanced cell migration, with the siKLF4 + TGF-β1 group showing the highest migration rate at 24 h in both macrophages and fibroblasts (Fig. 7C and D). These results indicate that KLF4 suppresses proliferation and migration in fibrotic contexts, and its loss may contribute to enhanced fibrotic activity.

## Discussion

This study performed unsupervised consensus clustering analysis using lipid-related genes, identifying two clusters associated with IPF. These clusters were significantly correlated with IPF-related lung function parameters, such as FVC and DLCO, as well as potential IPF-associated genes, including MUC5B, TGFβ1, SPP1, TERT, and COL3A1. Based on these clusters, a WGCNA was conducted, which identified a core module related to IPF, specifically the blue module. Key hub genes, including CAV1, KLF4, and AGTR1, were subsequently selected, as they exhibited high degrees in PPI analysis. Using these hub genes, diagnostic and prognostic models for IPF were constructed. Additionally, single-cell RNA sequencing analysis revealed high expression of KLF4 in alveolar epithelial cells, and cell experiments demonstrated that KLF4 knockdown promoted fibrosis in these cells.

The diagnostic model in this study was constructed using the backward stepwise generalized linear model (StepGLM) combined with gradient boosting machine (GBM). This approach demonstrated exceptional performance, achieving an AUC of over 0.95 in both the training and validation sets, which were derived from lung tissue samples. The high predictive accuracy can be attributed to the StepGLM’s ability to effectively select significant features and GBM’s strength in handling complex, non-linear relationships. Compared to previously reported models, the performance of this model is noteworthy. For example, Zhang et al. utilized a support vector machine algorithm to construct a predictive model for IPF, achieving AUC values of 0.893 and 0.851 in external validation datasets [44]. Similarly, He et al. developed a random forest diagnostic model based on circadian rhythm-related genes, reporting AUC values of 0.905 and 0.767 in internal and external validations, respectively [45]. Despite its strong performance, a limitation of this study is the exclusive use of lung tissue data, which may restrict the model’s generalizability to other sample types, such as blood or bronchoalveolar lavage fluid. Future studies should validate the model using diverse datasets and external samples to enhance its clinical applicability.

The prognostic model was constructed using bidirectional stepwise Cox regression (StepCox[both]) combined with the RSF algorithm, achieving an average AUC of 0.71 across the training and validation sets. The training set, derived from alveolar lavage fluid (OS), showed superior predictive performance compared to the blood-based validation sets (TFS and PFS). This discrepancy likely arises from the specificity of alveolar lavage fluid, which more directly reflects local pulmonary fibrosis processes, whereas blood samples, influenced by systemic factors, may dilute disease-specific markers. For example, Jing et al. developed a gene-based scoring system using PCA but did not provide AUC metrics for quantitative performance evaluation [46]. Besides, Lee et al. constructed a deep learning-based model using chest radiographs, achieving TD-AUCs ranging from 0.76 to 0.83 in external cohorts. However, reliance on imaging data in Lee’s model limits its applicability to settings with accessible radiographic resources [47]. By contrast, the StepCox[both] + RSF model combines the interpretability of Cox regression with the flexibility of machine learning, leveraging gene expression data to achieve consistent performance across different sample types.

In the present study, transcriptomic data from BALF and peripheral whole blood were integrated to develop and validate predictive models for IPF. Despite the marked differences in cellular composition between these two biospecimens—BALF being predominantly composed of alveolar macrophages and epithelial cells, and whole blood consisting of a heterogeneous mixture of circulating immune cells—the constructed models exhibited robust and reproducible predictive performance across datasets. Notably, the model trained on BALF samples achieved higher predictive accuracy when validated using blood-derived datasets, suggesting that BALF may better represent the pathophysiological changes occurring in the lung microenvironment. This observation is consistent with previous findings highlighting the utility of BALF in detecting early fibrotic changes and immune dysregulation specific to the lower respiratory tract [14, 48]. Although whole blood is convenient and non-invasive for clinical testing, these findings highlight the greater disease relevance of BALF for IPF biomarker discovery. Future studies should focus on collecting samples from multiple sources, especially lung tissue and BALF, to enhance the clinical utility and mechanistic understanding of transcriptomic IPF models.

This study also identified KLF4 as a potential pathogenic factor in IPF. KLF4 is a member of the evolutionarily conserved zinc finger transcription factor family, widely expressed in various tissues and cell types. It plays a critical role in regulating cell differentiation and is involved in numerous pathological processes, including inflammation and tumor progression [49]. Furthermore, KLF4 is one of the four core transcription factors essential for reprogramming somatic cells into induced pluripotent stem cells [50]. In vascular inflammation, estrogen induces the transdifferentiation of vascular smooth muscle cells into macrophage-like cells via KLF4, underscoring its role in cellular plasticity [51]. KLF4 is strongly upregulated in M2 macrophages while being significantly downregulated in M1 macrophages. Through cooperation with STAT6, KLF4 promotes the expression of M2-related genes and suppresses the production of pro-inflammatory cytokines [52]. In silicosis, vitamin D facilitates macrophage polarization toward the M2 phenotype via KLF4, thereby reducing inflammation in damaged tissues [53]. Similarly, low-dose decitabine enhances M2 macrophage polarization in patients with primary immune thrombocytopenia by increasing KLF4 expression [54]. Interestingly, a study on rheumatoid arthritis revealed that overexpression of KLF4 in macrophages led to an M1 phenotype, characterized by elevated inflammatory cytokine production. This shift exacerbated chondrocyte injury and apoptosis, promoting synovial tissue inflammation and joint damage [55].

The role of KLF4 in IPF has been increasingly recognized as critical for understanding disease pathogenesis and identifying potential therapeutic strategies. Several studies have demonstrated that KLF4 expression is significantly reduced in the lung tissues of IPF patients and in bleomycin-induced pulmonary fibrosis models. This downregulation correlates with exacerbated fibrosis and impaired cellular function in alveolar epithelial cells [56, 57]. Mechanistically, KLF4 regulates key signaling pathways involved in pulmonary fibrosis. One prominent mechanism involves the HIF-1α/endoplasmic reticulum stress pathway [56]. Another important mechanism is its ability to suppress epithelial-mesenchymal transition, a process pivotal to IPF pathogenesis. KLF4 overexpression attenuates TGF-β1-induced EMT by inhibiting Smad2/3 and Dvl signaling, whereas KLF4 knockdown promotes EMT and exacerbates fibrosis [58]. Inhibition of KLF4 expression has been linked to M2 macrophage polarization, which promotes profibrotic activity through enhanced TGF-β1 production.Targeting M2 polarization via agents like eucalyptol has been shown to regulate KLF4 and attenuate fibrosis, suggesting a broader impact of KLF4 across multiple cell types involved in IPF [59].

This study revealed the potential pathogenic role of KLF4 in IPF and successfully constructed diagnostic and prognostic models. However, the functional validation primarily focused on KLF4 expression changes and preliminary functional assays, lacking more in-depth mechanistic experiments. Studies using gene knockout models or complex in vivo experiments are needed to clarify how KLF4 regulates fibrosis-related signaling pathways at cellular and molecular levels. Additionally, while the diagnostic and prognostic models demonstrated strong performance, the data sources, including single-cell sequencing and in vitro experiments, were limited in sample size, especially in clinical samples, which may affect the generalizability of the findings. The reliance on lung tissue samples, while informative, may not fully reflect pathological processes in other biological samples, such as blood or bronchoalveolar lavage fluid, thereby limiting broader clinical applicability. Furthermore, integrating data from heterogeneous sources may have introduced inconsistencies, particularly due to variations across different databases and research platforms. The in vitro experiments relied on a TGF-β1-induced fibrosis model, which replicates certain aspects of fibrosis but does not fully capture the complex microenvironment of IPF in vivo. Additionally, factors such as oxidative stress, metabolic alterations, or immune microenvironment changes that could influence KLF4 function were not comprehensively investigated. Despite these limitations, the diagnostic and prognostic models provide a valuable framework for future studies and clinical applications.

## Conclusion

This study successfully constructed diagnostic and prognostic models for IPF, demonstrating strong predictive performance and clinical potential. Additionally, KLF4 was identified as a potential pathogenic factor, with its role in fibrosis-related processes highlighting its promise as a therapeutic target. Further validation and mechanistic studies are needed to enhance the clinical applicability of the models and to elucidate the molecular mechanisms of KLF4 in IPF.

## Electronic supplementary material

Below is the link to the electronic supplementary material.

Supplementary Material 1

Supplementary Material 2

Supplementary Material 3

Supplementary Material 4

Supplementary Material 5

Supplementary Material 6

Supplementary Material 7

## Data Availability

The data that support the findings of this study are available from the corresponding author, Caiyu Jiang, upon reasonable request.
