# Supplementary material for: Machine learning identifies lipid-associated genes and constructs diagnostic and prognostic models for idiopathic pulmonary fibrosis
Source: Orphanet J Rare Dis. 2025 Jul 10;20:354. doi: 10.1186/s13023-025-03876-0 (PMC12247251; doi:10.1186/s13023-025-03876-0)
Supplement: Supplementary file 1 — Supplementary Material 1 [file 13023_2025_3876_MOESM1_ESM.doc]

Supplementary table 1. PCR primer sequences.

| name | Forward | Reverse |
| --- | --- | --- |
| Col1 | 5′-GAGGGCCAAGACGAAGACATC-3′ | 5′-CAGATCACGTCATCGCACAAC-3′ |
| Col3 | 5′-GGAGCTGGCTACTTCTCGC-3′ | 5′-GGGAACATCCTCCTTCAACAG-3′ |
| α-SMA | 5′-AAAAGACAGCTACGTGGGTGA-3′ | 5′-GCCATGTTCTATCGGGTACTTC-3′ |
| KLF4 | 5′-CCCACATGAAGCGACTTCCC-3′ | 5′-CAGGTCCAGGAGATCGTTGAA-3′ |
| GAPDH | 5′-GGAGCGAGATCCCTCCAAAAT-3′ | 5′-GGCTGTTGTCATACTTCTCATGG-3′ |
| siRNA-KLF4 | 5′-GGUCAUCAGUGUUAGCAAAdTdT-3′ | 5′-UUUGCUAACACUGAUGACCdTdT-3′ |
