# Supplementary material for: Machine learning identifies lipid-associated genes and constructs diagnostic and prognostic models for idiopathic pulmonary fibrosis
Source: Orphanet J Rare Dis. 2025 Jul 10;20:354. doi: 10.1186/s13023-025-03876-0 (PMC12247251; doi:10.1186/s13023-025-03876-0)
Supplement: Supplementary file 2 — Supplementary Material 2 [file 13023_2025_3876_MOESM2_ESM.doc]

Supplementary table 2. lipid-related core genes in blue module.

| MMP1 |
| --- |
| COL7A1 |
| MSI2 |
| SPP1 |
| MMP7 |
| KRT17 |
| ADAMTS14 |
| CFB |
| STAR |
| COL17A1 |
| CDH3 |
| MMP10 |
| KCNN4 |
| SYT12 |
| GALNT14 |
| PAIP2B |
| CHRDL2 |
| ALDH1A3 |
| GLB1L3 |
| SLC2A5 |
| BDKRB2 |
| KRT6C |
| AK4 |
| NGFR |
| IVL |
| CABYR |
| SMOC1 |
| KCNG1 |
| ESR2 |
| SPRR1B |
| DEFB4A |
| LYPD1 |
| TTYH1 |
| RALGPS2 |
| RARRES1 |
| SALL4 |
| MMP3 |
| RAMP1 |
| KRT6B |
| ZNF215 |
| TIMP4 |
| HS6ST2 |
| C4B |
| TG |
| STAC2 |
| ASB2 |
| MMP12 |
| ATP10B |
| RNF175 |
| WNT6 |
| COL22A1 |
| KCNH8 |
| IL22RA2 |
| PTGES |
| GRHL3 |
| CCL7 |
| CASQ1 |
| CKMT2 |
| NMNAT2 |
| AVPR1A |
| SDS |
| OGDHL |
| DDIT4L |
| C15orf48 |
| MMP9 |
| SERPINA3 |
| AP3B2 |
| MS4A6E |
| AHNAK2 |
| CATSPERB |
| TM4SF19 |
| WNT10A |
| KRT75 |
| C1QL2 |
| SPRR2D |
| CNIH3 |
| GSC |
| APOD |
| BNC1 |
| SLC13A5 |
| NPFFR2 |
| PAEP |
