# Supplementary material for: Machine learning identifies lipid-associated genes and constructs diagnostic and prognostic models for idiopathic pulmonary fibrosis
Source: Orphanet J Rare Dis. 2025 Jul 10;20:354. doi: 10.1186/s13023-025-03876-0 (PMC12247251; doi:10.1186/s13023-025-03876-0)
Supplement: Supplementary file 3 — Supplementary Material 3 [file 13023_2025_3876_MOESM3_ESM.doc]

Supplementary table 3. Features of the diagnostic model.

| CRTAC1 |
| --- |
| ITLN2 |
| KCNMB4 |
| CEACAM21 |
| ANO4 |
| ADAMTSL3 |
| ALOX12 |
| CACNA2D2 |
| ARSE |
| ANKRD1 |
| CDO1 |
| CYP2C18 |
| IGFALS |
| CLEC4M |
| ESYT3 |
