# Supplementary material for: Machine learning identifies lipid-associated genes and constructs diagnostic and prognostic models for idiopathic pulmonary fibrosis
Source: Orphanet J Rare Dis. 2025 Jul 10;20:354. doi: 10.1186/s13023-025-03876-0 (PMC12247251; doi:10.1186/s13023-025-03876-0)
Supplement: Supplementary file 4 — Supplementary Material 4 [file 13023_2025_3876_MOESM4_ESM.doc]

Supplementary table 4. Features of the prognostic model.

| ANXA3 |
| --- |
| ANKRD29 |
| GPA33 |
| CYP3A7 |
| KL |
| DISP1 |
| LIFR |
| CLIC5 |
| HBEGF |
| CYP2C18 |
