# Supplementary material for: Machine learning identifies lipid-associated genes and constructs diagnostic and prognostic models for idiopathic pulmonary fibrosis
Source: Orphanet J Rare Dis. 2025 Jul 10;20:354. doi: 10.1186/s13023-025-03876-0 (PMC12247251; doi:10.1186/s13023-025-03876-0)
Supplement: Supplementary file 5 — Supplementary Material 5 [file 13023_2025_3876_MOESM5_ESM.doc]

Supplementary table 5. The degree of genes in the PPI network.

| id | Degree |
| --- | --- |
| CAV1 | 8 |
| KLF4 | 6 |
| AGTR1 | 5 |
| KLRD1 | 4 |
| CYP3A5 | 4 |
| FCN3 | 4 |
| KLRF1 | 3 |
| GNLY | 3 |
| FGFBP2 | 3 |
| ID1 | 3 |
| EPAS1 | 3 |
| LIFR | 3 |
| EDNRB | 2 |
| ACVRL1 | 2 |
| CD5L | 2 |
| CLEC4M | 2 |
| CYP3A7 | 2 |
| CYP2C18 | 2 |
| CAV2 | 2 |
| KLF2 | 2 |
| CDKN2B | 2 |
| C4BPA | 2 |
| F11 | 2 |
| CCRL2 | 2 |
| LEFTY2 | 2 |
| APOH | 2 |
| IL1RL1 | 2 |
| DKK2 | 1 |
| ADCY8 | 1 |
| EGR2 | 1 |
| CHRM3 | 1 |
| ADRA1A | 1 |
| HSD17B6 | 1 |
| HIF3A | 1 |
| HBEGF | 1 |
| IGFALS | 1 |
| DISP1 | 1 |
| IL17RB | 1 |
| AFF2 | 1 |
| GBP1 | 1 |
| IFIT2 | 1 |
| DPP6 | 1 |
| GPM6A | 1 |
| AFF3 | 1 |
