# Supplementary material for: Machine learning identifies lipid-associated genes and constructs diagnostic and prognostic models for idiopathic pulmonary fibrosis
Source: Orphanet J Rare Dis. 2025 Jul 10;20:354. doi: 10.1186/s13023-025-03876-0 (PMC12247251; doi:10.1186/s13023-025-03876-0)

Supplementary Figure 1. Single-cell transcriptomic analysis of lung samples from IPF and control groups. (A) Dot plot showing the expression patterns of canonical marker genes across major cell types, including airway epithelium, alveolar type II cells, alveolar type I cells, and macrophages. Dot size indicates the percentage of cells expressing each gene, and color intensity represents average expression. (B) UMAP plot showing the unsupervised clustering and automated annotation of major cell types based on transcriptomic features. (C) Proportions of cells with high and low KLF4 expression in three representative cell types (alveolar type II cells, fibroblasts, and macrophages) across control and IPF groups.
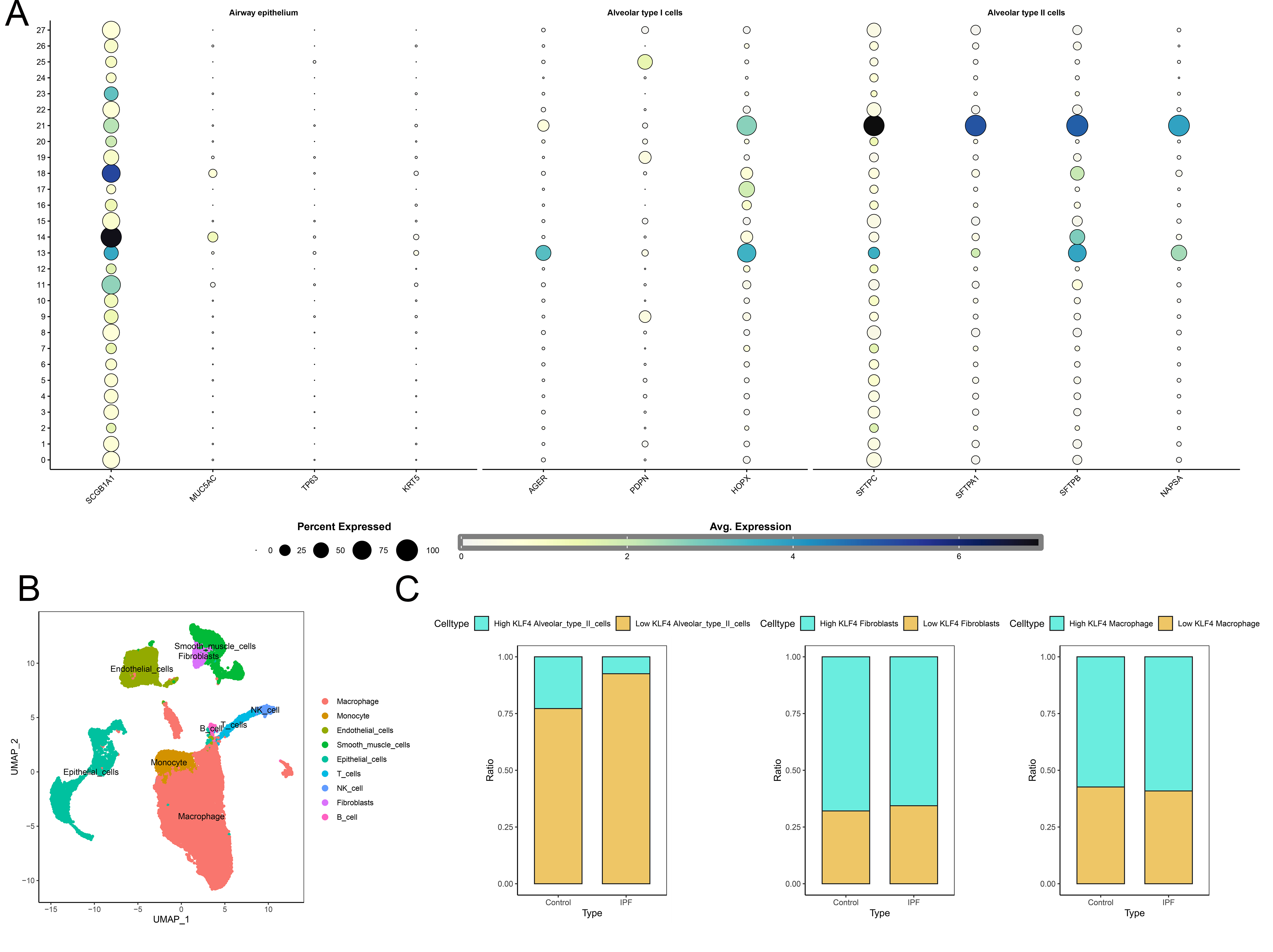

Supplement: Supplementary file 6 — Supplementary Material 6 [file 13023_2025_3876_MOESM6_ESM.doc]
