# Supplementary material for: Machine learning identifies lipid-associated genes and constructs diagnostic and prognostic models for idiopathic pulmonary fibrosis
Source: Orphanet J Rare Dis. 2025 Jul 10;20:354. doi: 10.1186/s13023-025-03876-0 (PMC12247251; doi:10.1186/s13023-025-03876-0)

Supplementary Figure 2. Lipid Metabolism Activity in IPF. (A) Overall lipid metabolism module scores in control vs. IPF samples. Scores were calculated using the AddModuleScore function based on lipid-associated gene sets. A significant reduction in lipid metabolism activity was observed in IPF. (B) Lipid metabolism scores across different cell types in the IPF group, highlighting cell type–specific metabolic differences.


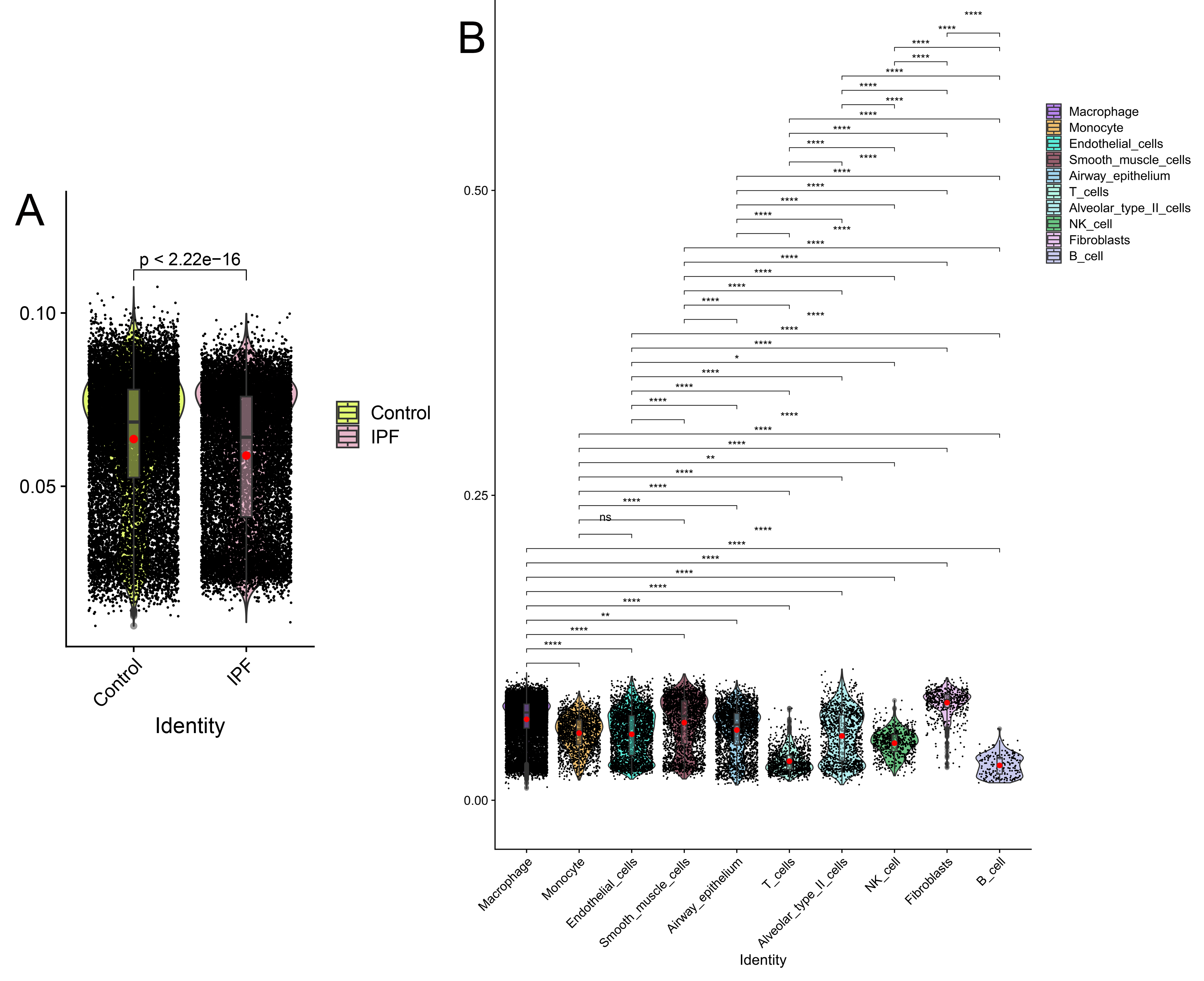

Supplement: Supplementary file 7 — Supplementary Material 7 [file 13023_2025_3876_MOESM7_ESM.doc]
